# Effects of Short-Term Dynamic Constant External Resistance Training and Subsequent Detraining on Strength of the Trained and Untrained Limbs: A Randomized Trial

**DOI:** 10.3390/sports4010007

**Published:** 2016-01-27

**Authors:** Pablo B. Costa, Trent J. Herda, Ashley A. Herda, Joel T. Cramer

**Affiliations:** 1 Exercise Physiology Laboratory, Department of Kinesiology, California State University, Fullerton, CA 92831, USA; 2 Department of Health, Sport and Exercise Sciences, University of Kansas, Lawrence, KS 66045, USA; t.herda@ku.edu (T.J.H.); a.herda@ku.edu (A.A.H.); 3 Department of Nutrition and Health Sciences, University of Nebraska—Lincoln, Lincoln, NE 68583, USA; jcramer@unl.edu

**Keywords:** training-induced, neuromuscular adaptation, isotonic, muscle mechanics, unilateral, cross education

## Abstract

Short-term resistance training has been shown to increase isokinetic muscle strength and performance after only two to nine days of training. The purpose of this study was to examine the effects of three days of unilateral dynamic constant external resistance (DCER) training and detraining on the strength of the trained and untrained legs. Nineteen men were randomly assigned to a DCER training group or a non-training control group. Subjects visited the laboratory eight times, the first visit was a familiarization session, the second visit was a pre-training assessment, the subsequent three visits were for training sessions (if assigned to the training group), and the last three visits were post-training assessments 1, 2, and 3 (*i.e.*, 48 h, 1 week, and 2 weeks after the final training session). Strength increased in both trained and untrained limbs from pre- to post-training assessment 1 for the training group and remained elevated at post-training assessments 2 and 3 (*p* ≤ 0.05). No changes were observed in the control (*p* > 0.05). Possible strength gains from short-term resistance training have important implications in clinical rehabilitation settings, sports injury prevention, as well as other allied health fields such as physical therapy, occupational therapy, and athletic training.

## 1. Introduction

Allied health professionals, such as physical therapists, occupational therapists, and athletic trainers, may benefit from rapid increases in strength of a patient or athlete recovering from injury [1,2,3]. In theory, if an individual’s strength can be increased within a short period of time, an alternative to more expensive and invasive medical procedures may be offered [1,2]. In addition, they are more likely to comply with a rehabilitation program and perhaps decrease the risk of reinjury [3]. Consequently, short-term resistance training has been shown to increase isokinetic muscle strength and performance after only two to nine days of training [1,2,4,5]. This short time course for strength adaptations may conveniently coincide with the commonly limited rehabilitation treatments due to minimal insurance coverage or lack of compliance [1,2], or the time demands for return to play in sports. If patients do not improve quickly, the risk of injury reoccurrence may increase [1]. This potential for short-term resistance training to improve muscular performance in a relatively shorter period of time would have important implications for professionals working in rehabilitation settings [1,2,3].

Evidence has shown that improvements in muscle performance can be observed in a shorter period than what is typically used in longer traditional training programs [1,2,6,7]. For example, Prevost *et al.*, (1999) investigated velocity-specific short-term training for two days and reported 22.1% increases in peak torque (PT) at 270°∙s^−1^ after training at 270°∙s^−1^, but no changes for training at 30 and 150°∙s^−1^ at the testing velocities of 30 and 150°∙s^−1^ [4]. Similarly, Coburn *et al.*, (2006) compared short-term resistance training effects after three sessions of slow- or fast-velocity and found that PT increased for both training groups [2]. However, the slower velocity training group increased PT at both velocities whereas PT increased only at the faster velocity for the faster velocity training group [2]. No changes in PT were observed for the control group and no changes in EMG amplitude were reported for any of the groups at any of the velocities. The authors concluded three sessions of slow or fast velocity isokinetic resistance training were sufficient to increase PT and the lack of EMG amplitude changes suggested increases in leg extension PT were not caused by increases in muscle activation [2].

The principle of training called reversibility, or detraining, occurs when a complete cessation or substantial reduction in training causes a partial or complete reversal of the adaptations induced by training [8,9]. Detraining occurs after an individual discontinues a training program [8,9,10,11,12,13,14,15]. Most of the increases in strength found with resistance training are lost after several weeks of detraining [10,11,12,13,14,16,17]. However, Colliander and Tesch (1992) showed that a resistance training program incorporating combined concentric and eccentric leg extension exercise retained more of the novel strength gains than a concentric-only training program [16]. In addition, Farthing (2003) found eccentric muscle action training elicited greater strength gains than concentric training [18]. Because isokinetic muscle actions are typically concentric, it is unknown whether dynamic constant external resistance (DCER) training, which uses coupled concentric and eccentric muscle actions, and isokinetic training would affect detraining differently.

Isokinetic muscle actions have been traditionally used in rehabilitation and testing scenarios. Several studies have examined the effects of isokinetic training on strength and/or muscle cross-sectional area (CSA) [1,2,4,5] and isokinetic training allows development of maximum tension throughout the range of motion [7]. However, DCER training would offer a more accessible, convenient, cost-effective, and practical method of training, in addition to perhaps providing a greater stimulus to elicit increases in strength [19]. Furthermore, no studies have investigated the effects of short term resistance training on the contralateral untrained limb or on detraining. Therefore, the purpose of this study was to examine the effects of three days of DCER training and subsequent detraining on isokinetic on strength of the trained and untrained contralateral leg extensors during maximal leg extension muscle actions.

## 2. Method

### 2.1. Subjects

Nineteen apparently healthy untrained men (mean ± SD age = 21.6 ± 3.4 years; body mass = 77.9 ± 14.0 kg; height = 173.9 ± 4.1 cm) were randomly assigned to a DCER training group or control group. Participants were minimally active and naïve to the intent of the study. Individuals with a history of chronic resistance training (>1 day/week) in the previous 12 months or those who reported engaging in one or more lower-body resistance training exercise for six months prior to the start of the study were excluded from participating. Prior to any testing, all subjects read and signed an informed consent form and completed a health status questionnaire. Individuals with any degenerative neuromuscular or joint disorders, or who sustained injuries distal to the waist within six months prior to screening were also excluded from the study. Subjects were asked to maintain their daily activities, but refrain from any exercise and/or nutritional supplements throughout the course of the study. Individuals who had been taking nutritional supplements three months prior to screening were not permitted to participate. This study was approved by the university’s Institutional Review Board for the Protection of Human Subjects.

### 2.2. Research Design

A mixed factorial design was used to examine the effects of three days of short-term unilateral resistance training and subsequent detraining on strength. Subjects visited the laboratory on eight separate occasions. The first visit was a familiarization session, the second visit was a pre-training assessment, the subsequent three visits were for training (if assigned to the training group), and the last three visits were the post-training assessments (*i.e.*, 48 h, 1 week, and 2 weeks after the final training session). Pre-training assessments were performed 48 h prior to the start of training. Testing included assessments of DCER strength. The training group performed DCER leg extension exercise with the dominant leg in each of the three days of training whereas the control group did not take part in the training intervention. After the three training sessions, post-training assessments were performed in an identical manner to the pre-training assessments. In order to examine the time course of the effects of training, post-training assessments were performed 48 h, 1 week, and 2 weeks after the final training session. All pre- and post-training assessments were conducted at approximately the same time of day.

### 2.3. Dynamic Constant External Resistance Assessments

The maximal strength of the leg extensors were assessed using a DCER Nautilus leg extension machine (Nautilus, Inc., Vancouver, WA, USA). The input axis of the machine was aligned with the axis of rotation of the knee. The distal anterior portion of the leg superior to the ankle was used as the load bearing point. Three submaximal warm-up sets of increasing tester-selected intensities (*i.e.*, 6–8, 3–5, and 1–2 repetitions) preceded the maximal strength attempt. When one attempt was successful, the load was increased by 2–5 kg until a failed repetition occurred. A failed repetition was defined as the inability to complete the full range of motion with the assigned load. During the tests, loud verbal encouragement was provided by the investigator. Each subject was instructed to provide maximal effort throughout the entire range of motion. The greatest load moved through a complete leg extension range of motion was considered the one repetition maximum (1-RM). A 1-min rest was allowed between each successive attempt [20,21].

### 2.4. Dynamic Constant External Resistance Training Protocol

After a rest period of 48 h following the pre-training assessment, the training group took part in three DCER training sessions separated by 48 h. Participants in the training group performed 4 sets of 10 repetitions. Each training session began with ten warm-up repetitions at approximately 25% of the resistance used for the DCER training session. Approximately 80% of the 1-RM obtained during the DCER maximal strength assessment was used as the starting load for the DCER group. A 2-min rest period was allowed between each training set. Training load for the DCER group was continually increased and adjusted by approximately 1.14 kg as each participant was able to tolerate a given load with ease in order to ensure that the subject reached failure at approximately the 10th repetition. All participants taking part in the DCER training intervention were supervised during all training sessions.

### 2.5. Rating of Perceived Exertion

Rating of perceived exertion (RPE) was used to compare effort among the DCER training days and sets [22,23,24,25,26]. Prior to the start of the study, subjects received instructions on how to use the RPE scale to rate their perceived exertion. A Category-Ratio scale (CR-10) was used, where “0” is classified as rest (no effort) and “10” is classified as maximal effort (most stressful exercise ever performed). The CR-10 has been slightly modified to reflect American English (e.g., easy and hard instead of light and strong, respectively) [24]. Subjects were asked to provide a number on the scale to rate their overall effort immediately after each set was completed and after the entire training session. The RPE assessments were conducted during each session by showing the scale and asking subjects “How would you rate your effort?” and “How would you rate your entire workout?” immediately after each set of training and after each training session, respectively. Therefore, in this study, “set RPE” was defined as the RPE reported by the subject after each set, while “session RPE” was defined as the RPE reported each day after the training session was completed.

### 2.6. Statistical Analyses

A three-way mixed factorial ANOVA (time (pre- *vs.* post-training assessment 1 *vs.* post-training assessment 2 *vs.* post-training assessment 3) × group (DCER *vs.* control) × limb (trained *vs.* untrained) was used to analyze the 1-RM data. A two-way repeated measured ANOVA (time [training session 1 *vs.* training session 2 *vs.* training session 3) × set (1 *vs.* 2 *vs.* 3 *vs.* 4)) was used to analyze RPE assessed after each set during training. A one-way repeated measures ANOVA (time (training session 1 *vs.* training session 2 *vs.* training session 3)) was used to analyze training session RPE. When appropriate, follow-up analyses were performed using lower-order two- and one-way repeated measured ANOVAs, and paired sample *t*-tests. An alpha level of *p* ≤ 0.05 was considered statistically significant for all comparisons. Predictive Analytics SoftWare (PASW) version 18.0.0 (SPSS Inc., Chicago, IL, USA) was used for all statistical analyses.

## 3. Results

### 3.1. Dynamic Constant External Resistance Assessments

**Figure 1:**
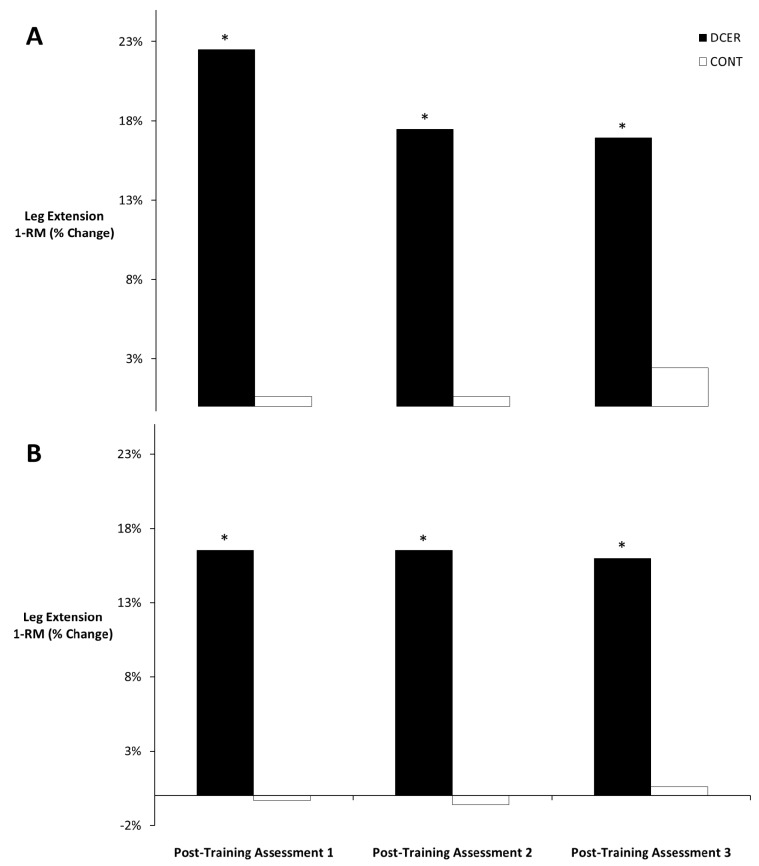
Means of percent change for leg extension 1-RM for the trained (**A**) and untrained (**B**) legs. * Denotes significant difference from the pre-test for the DCER group. DCER = dynamic constant external resistance; CONT = control.

**Figure 2:**
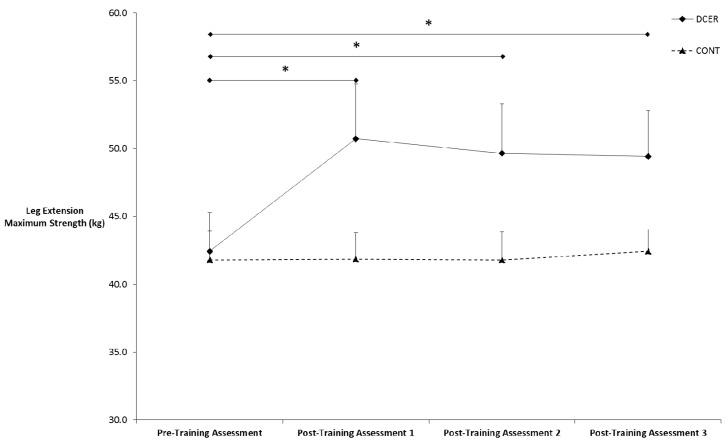
Means (±SE) for leg extension 1-RM collapsed across limb. * Denotes significant difference from the pre-test for the DCER group. DCER = dynamic constant external resistance; CONT = control.

Table 1 contains the means (±SE) for 1-RM strength in the trained and untrained leg. There was no three-way interaction for time × group × limb (*p* = 0.11). However, there was a significant two-way interaction for time × group (*p* = 0.001). Post-hoc pairwise comparisons for the marginal means indicated that 1-RM increased in both trained and untrained limbs from pre- to post-training assessment 1 for the DCER group (*p* < 0.001) (Figure 1). There were no differences in 1-RM strength for the DCER group among post-training assessments 1, 2, and 3 (*p* > 0.05) (Figure 2). No significant changes were found for the control group (*p* > 0.05).

**Table 1:** Means (±SE) for leg extension 1-RM.

Group			Pre-Training Assessment 1	Post-Training Assessment 1	Post-Training Assessment 2	Post-Training Assessment 3
1-RM (kg)	DCER (*n* = 10)	Trained	43.0 ± 3.0	52.6 ± 3.8 *	50.5 ± 3.5 *	50.2 ± 3.2 *
		Untrained	41.9 ± 2.7	48.9 ± 4.2 *	48.9 ± 3.8 *	48.6 ± 3.5 *
	CONT (*n* = 9)	Trained	41.7 ± 2.2	41.9 ± 2.1	41.8 ± 1.9	42.7 ± 1.6
		Untrained	41.9 ± 2.1	41.8 ± 1.9	41.7 ± 2.0	42.2 ± 1.7

Notes: 1-RM = 1 repetition maximum; DCER = dynamic constant external resistance; CONT = control. * Denotes significant change from pre- to post-assessments.

### 3.2. Rating of Perceived Exertion

Table 2 contains the means (±SE) for set and session RPE from the training group. There was no two-way interaction for time × set for set RPE (*p* = 0.41). However, there was a significant main effect for set (*p* < 0.001). Post-hoc pairwise comparisons for the marginal means (collapsed across time) indicated a significant main effect for set RPE (*p* < 0.05). RPE increased significantly from the first until the last set within each session (*p* < 0.05). For session RPE, there was no main effect for time (*p* = 0.55).

**Table 2:** Means (±SE) for set and session rating of perceived exertion for the DCER group.

Training Session	1st Set	2nd Set	3rd Set	4th Set	Session
Session 1	6.4 ± 0.54	7.3 ± 0.63 *	8.3 ± 0.45 *	8.6 ± 0.37 *	7.6 ± 0.48
Session 2	5.4 ± 0.37	6.9 ± 0.31 *	7.8 ± 0.29 *	8.6 ± 0.43 *	7.1 ± 0.35
Session 3	5.8 ± 0.33	6.9 ± 0.43 *	7.9 ± 0.50 *	8.5 ± 0.48 *	7.5 ± 0.40

Notes: DCER = dynamic constant external resistance. * Denotes significant change over sets within each training session.

## 4. Discussion

Perhaps the most important finding of the present study was the increase in DCER strength acquired by the training group. DCER strength increased from pre- to post-training assessment 1 in the trained and untrained legs for the DCER training group and remained elevated during post-training assessments 2 and 3. To our knowledge, this was the first study to report DCER strength gains with short-term resistance training while also considering the detraining period of two weeks. These findings are in agreement with previous studies reporting PT increases after short-term isokinetic training [1,2]. In addition, the DCER group retained the strength gains during post-training assessments 2 and 3. That is, DCER strength remained elevated over a two-week period. Typical increases in strength obtained in longer resistance training programs are diminished after several weeks of detraining [10,11,12,13,14,16]. Colliander and Tesch (1992) compared the effects of resistance training and detraining using concentric-only and combined concentric and eccentric muscle actions of the leg extensors and reported that the group performing coupled concentric and eccentric muscle actions had a greater overall increase in PT after training and detraining than the concentric-only group [16]. These authors suggested strength decreases observed during detraining are not likely due to atrophy, but perhaps a reduction in neural drive or motor unit activation and hypothesized eccentric muscle actions are capable of inducing greater motor unit activation than concentric muscle actions [16]. Thus, it was suggested a resistance training program incorporating combined concentric and eccentric repetitions of leg extension can retain more of the obtained strength gains than the training program with concentric-only repetitions [16]. Likewise, Farthing (2003) found eccentric-only muscle action training elicited greater strength gains than concentric-only training [18]. Similarly, Knight *et al.*, (2001) suggested that isotonic muscle actions may be more effective at increasing torque because isokinetic resistance is accommodating, hence, it decreases with fatigue [19]. These findings [16,18,19], along with the findings of the current study may indicate an advantage of DCER over isokinetic resistance training programs when conducted over a relatively short period of time.

For the DCER training group, despite training only one leg, strength increased on the contralateral limb and was maintained over the two-week detraining period. Unilateral resistance training of a limb can increase the strength of the contralateral limb through a concept termed cross-education [27]. Increases in strength of the contralateral, untrained limb, have been extensively reported in the literature [27,28]. Possibly an important finding of the current study is that short-term resistance training also elicited the cross-education effect. This has important implications for injury rehabilitation, where in the initial period post-injury strength gains on an injured limb can conceivably be obtained with short-term contralateral resistance training. Contralateral strength gains have been hypothesized to be attributed to central neural adaptations (*i.e.*, excitation of the cortex), increased motoneuron output, and improved postural stabilization [27,28,29]. Accordingly, structural changes in the brain have been reported after only four weeks of unilateral resistance training concomitant with strength increases in trained and untrained limb [30]. In fact, strength gains may not be restricted to the contralateral untrained muscle, but might be observed in the contralateral untrained antagonist muscle [31]. Therefore, future studies should investigate the effects of short-term resistance training on contralateral antagonist muscles.

Strength gains were also maintained during the two-week detraining period in the DCER group. Although in the present study subjects were untrained, these findings were similar to those of Hortobagyi *et al.*, (1993), who found that two weeks of detraining of resistance-trained athletes did not cause a significant decrease in maximal bench press, squat, isometric, or concentric isokinetic strength [32]. Similarly, Shaver (1975) reported that recently acquired strength can be maintained in both trained and untrained limb for up to one week [33]. To our knowledge, the current study is the first to demonstrate short-term increases in strength can be maintained for a two-week period and in untrained limbs. In contrast, other authors have suggested strength gains that have been recently acquired may diminish faster than in strength-trained athletes [9,33]. Thus, the experience with resistance training (novice *vs.* well-trained athletes) should be considered when interpreting the results of a short-term resistance training program and its potential lasting effects.

The neuromuscular system undergoes numerous adaptations following a resistance training program [6,7,34,35,36,37,38]. Short-term resistance training has been shown to increase muscle strength and isokinetic performance after only a few days of training. Increases in muscular strength following a resistance training program can be attributed to neural and hypertrophic factors [6,34,35,36,37,39]. Therefore, voluntary strength increases due to not only the CSA and quality of muscle mass but also to the extent in which the muscle mass is able to activate [39]. In general, neural factors are believed to account for most of the increases in strength in the early stages of a resistance training program, whereas hypertrophic factors gradually become prevalent after several weeks of training [6,36,38,39,40,41,42]. Research suggests early adaptations to resistance training programs are related to improvements in neuromuscular efficiency, which perhaps indicates an increased capacity to activate skeletal muscle voluntarily [1,2,4,7,42]. Hence, initial improvements in strength and muscular performance reported following short-term resistance training are generally attributed to neural adaptations rather than muscle fiber hypertrophy [6,7]. However, the specific mechanisms of such adaptations in short-term training are not fully understood [2]. For example, Akima *et al.*, (1999) reported increases in PT after two weeks of resistance training but no changes in muscle CSA or fiber area suggesting strength increases occurred without muscle hypertrophy [7]. Similarly, Prevost *et al.*, (1999) reported velocity-specific increases in PT training at 270°∙s^−1^ after increases in PT after two days of isokinetic training but not with training at 30 and 150°∙s^−1^ [4]. Because improvements were only seen in one velocity, and muscle hypertrophy would most likely yield strength increases at the other velocities, investigators suggested that neural adaptations play a major role in strength improvements which are specific to a training velocity [4]. Beck *et al.*, (2007) suggested that responses to training might be influenced by the number of training sessions, training volume, and muscle(s) being tested [3]. Nevertheless, Akima *et al.*, (1999) and Costa *et al.*, (2013) suggested future studies should investigate the precise mechanisms underlying strength gains obtained with short-term resistance training [7,43].

The results revealed there were no differences in RPE as acknowledged by the subjects among the DCER training sessions. However, RPE increased from the first to the fourth set within each training session. These results are similar to those found by Egan *et al.*, (2006), who reported mean session RPE values of 7.3 for six sets of six repetitions of traditional resistance training using squats at an intensity of 80% of 1-RM [22]. Likewise, Sweet *et al.*, (2004) reported mean RPE values between 6.8 and 8.2 for 70 and 90% of leg press 1-RM, respectively [23]. Thus, perceived effort from a short-term resistance training program in the current study was similar to previous studies and was not lower because of the shorter training program duration.

## 5. Conclusions

The primary finding of this study was that DCER strength increased in the trained and untrained limbs with three days of contralateral training. This has important implications for injury rehabilitation, where in the initial period post-injury, strength gains on an injured limb can possibly be obtained with short-term resistance training. Furthermore, research has shown the feasibility and benefits of preoperative resistance training prior to surgical intervention to decrease the odds of inpatient rehabilitation, reduce the length of hospital stay, and promote overall postoperative recovery [44,45,46,47]. It is believed the increases were due to an unidentified factor because of strength gains observed in the untrained limb after DCER resistance training. Future studies should investigate the precise physiological components responsible for short-term contralateral strength gains. The findings of the current study may indicate an advantage of DCER over isokinetic resistance training programs when conducted over a relatively short period of time. These findings have important implications in clinical rehabilitation settings, sports injury prevention, as well as in other allied health fields such as physical therapy, occupational therapy, and athletic training. To our knowledge, the current study is the first to demonstrate recently-acquired strength can be maintained for a two-week period in untrained limbs. Therefore, future studies should examine the effects of short-term resistance training on injury prevention and rehabilitation.
